# Targeting immunometabolism during cardiorenal injury: roles of conventional and alternative macrophage metabolic fuels

**DOI:** 10.3389/fphys.2023.1139296

**Published:** 2023-04-28

**Authors:** Alan J. Mouton, Jussara M. do Carmo, Alexandre A. da Silva, Ana C. M. Omoto, John E. Hall

**Affiliations:** ^1^ Department of Physiology and Biophysics, University of Mississippi Medical Center, Jackson, MS, United States; ^2^ Mississippi Center for Obesity Research, University of Mississippi Medical Center, Jackson, MS, United States

**Keywords:** heart, macrophage, kidney, inflammation, cardiac injury, renal injury, ketone, lactate

## Abstract

Macrophages play critical roles in mediating and resolving tissue injury as well as tissue remodeling during cardiorenal disease. Altered immunometabolism, particularly macrophage metabolism, is a critical underlying mechanism of immune dysfunction and inflammation, particularly in individuals with underlying metabolic abnormalities. In this review, we discuss the critical roles of macrophages in cardiac and renal injury and disease. We also highlight the roles of macrophage metabolism and discuss metabolic abnormalities, such as obesity and diabetes, which may impair normal macrophage metabolism and thus predispose individuals to cardiorenal inflammation and injury. As the roles of macrophage glucose and fatty acid metabolism have been extensively discussed elsewhere, we focus on the roles of alternative fuels, such as lactate and ketones, which play underappreciated roles during cardiac and renal injury and heavily influence macrophage phenotypes.

## 1 Introduction

Macrophages play critical roles in the response to injury in acute and chronic cardiac and renal diseases. Macrophages are necessary for driving reparative fibrosis and scar tissue formation during acute injury, but may perpetuate reactive fibrosis and dysfunction during chronic injury (Cao et al., 2015; Dutta and Nahrendorf, 2015; Justin Rucker and Crowley, 2017; Hulsmans et al., 2018; Mouton et al., 2018; Engel and Chade, 2019; Han et al., 2019; Mouton et al., 2020; Cantero-Navarro et al., 2021; Chen et al., 2022). Cardiac and renal injury are often accompanied by changes in tissue metabolism. Recent evidence suggests that metabolic shifts drive critical processes and gene expression in macrophages, which are influenced by substrate availability and preference (Ayala et al., 2019; Batista-Gonzalez et al., 2019; Bezold et al., 2019; Jing et al., 2020; Mouton et al., 2020; Geeraerts et al., 2021; Jiang et al., 2022; Matsuura et al., 2022). In this brief review, we discuss the basic roles of macrophages during these types of injury, and review the literature on immunometabolic shifts in the context of changes in whole tissue metabolism during injury. Finally, we discuss metabolic disorders (e.g., obesity, diabetes) which may alter substrate availability/preference and thus the immunometabolic response to injury.

## 2 Roles of macrophages during cardiac injury

### 2.1 Acute injury

Myocardial infarction (MI) affects ∼1 million United States citizens each year (Mouton et al., 2018; DeBerge et al., 2019; Mouton et al., 2020). Remodeling of the left ventricle after MI occurs in three phases: inflammation, proliferative/granulation, and scar maturation. Macrophages play critical roles in all three phases of the post-MI remodeling process (Ma et al., 2018). During the inflammatory phase, lymphocyte antigen 6C (Ly6C) expressing monocytes are attracted to the infarct region via gradients of chemokines, particularly CCL2 and CCL7, and then activated by exposure to damage-associated molecular patterns (DAMPs) in the heart (Peet et al., 2020). A much smaller second wave of Ly6C-low monocytes is also recruited to the heart, a process that depends on the CX3CR1 chemokine (Dutta and Nahrendorf, 2015). Tissue resident macrophages, which prevent adverse remodeling, are mostly depleted during this initial inflammatory phase (Bajpai et al., 2019; Dick et al., 2019). During the proliferative phase, pro-inflammatory macrophages (M1) derived from Ly6C-high monocytes begin to undergo differentiation into anti-inflammatory macrophage subsets (M2), while surviving resident M2 macrophages proliferate and begin to repopulate (Heidt et al., 2014; Ma et al., 2018). The scar maturation phase is characterized by reparative fibrosis of the infarcted area, which forms an organized tensile collagenous scar capable of preventing rupture (Dutta and Nahrendorf, 2015; DeBerge et al., 2019; Mouton et al., 2020). As the scar fully matures, infarct macrophages are almost completely derived from resident sources, assuming an M2 phenotype by secreting mediators such as transforming growth factor-beta-1 (TGFβ1) and interleukin (IL)-10 to promote myofibroblast differentiation and maintain immune quiescence (Heidt et al., 2014; Sager et al., 2016; Mouton et al., 2018). After MI, the remote non-infarcted zone also undergoes remodeling, typically at a much later time point. This is due to increased wall stress and compensatory workload, leading to hypertrophy, inflammation, and fibrosis, and ultimately development of heart failure in ∼50% of MI patients (Sutton and Sharpe, 2000). In summary, macrophages play a critical role in the early response to MI, and are mainly derived from infiltrating monocytes.

### 2.2 Chronic injury

While the roles of macrophages have been heavily emphasized in acute ischemic cardiac injury, less is known about their roles during chronic cardiac injury, such as occurs with hypertension/pressure overload and heart failure with preserved ejection fraction (HFpEF). However, recent studies have shed new light on the roles of macrophages during chronic cardiac injury. Significant macrophage expansion occurs after transverse aortic constriction (TAC) in mice, peaking at 1 week and returning to baseline levels by 2 weeks (Revelo et al., 2021). There is some controversy over which subpopulations characterize this initial macrophage expansion, as some investigators have reported that a Ly6Clow population predominates, while others have reported that Ly6C-high monocytes predominate (Weisheit et al., 2014; Patel et al., 2018). In any case, after 4–6 weeks of TAC, a second wave of Ly6Chigh monocytes begin to expand in the bone marrow and spleen before infiltrating the heart and causing deterioration of cardiac function (Liao et al., 2018; Bizou et al., 2021). As in the case of acute injury, resident macrophages appear to play a protective/reparative role during chronic injury, as depletion of resident macrophages with a CD115 blocking antibody exacerbates fibrosis and stimulates angiogenesis.

In addition to chronic conditions associated with impaired systolic function, macrophages may also play a role in heart failure with preserved ejection fraction (HFpEF) (Hulsmans et al., 2018; DeBerge et al., 2019). Cardiac macrophage proliferation is observed in HFpEF patients, and macrophage-derived IL-10 promotes cardiac fibrosis in a mouse model of HFpEF, indicating that excessive M2 activation may contribute to HFpEF (Hulsmans et al., 2018). Conversely, high fat diet-induced diastolic dysfunction is associated with expansion of M1 macrophages, which can be attenuated by depleting macrophages or administering an IL-1 receptor antagonist (Liu et al., 2023). However, further studies are needed to investigate other potential mechanisms by which macrophages contribute to HFpEF, such as the ability of pro-inflammatory cytokines released by macrophages to contribute to endothelial dysfunction, which impairs nitric oxide-mediated cardiomyocyte relaxation (Paulus and Tschope, 2013; Paulus and Zile, 2021; Schiattarella et al., 2022).

## 3 Roles of macrophages during renal injury

### 3.1 Acute injury

Acute kidney injury (AKI) is caused by a variety of insults, including sepsis, hypoxia, or hypovolemic shock, that may result in rapid decline of kidney function (Han et al., 2019). The injured kidney can be repaired and regenerated, depending on the extent of the injury, but severely injured renal tissue is ultimately replaced by fibrotic tissue, leading to permanent declines in renal function (Chen et al., 2022). Much like the heart, circulating monocytes infiltrate the injured kidney in response to DAMPs and chemokine gradients, and differentiate towards M1-like macrophages (Chen et al., 2022). Although an acute inflammatory response is necessary for repair/regeneration of the injured kidney, excessive or prolonged M1 macrophage activation (i.e., due to heightened inflammation in T2D or obese patients) can exacerbate injury due to the actions of inflammatory cytokines, which induce further glomerular and tubular injury by promoting apoptosis of parenchymal cells and driving fibrosis (Cao et al., 2015). M1 macrophages can also cause endothelial dysfunction, leading to increased glomerular pressures and ultimately further injury and additional impairment of glomerular filtration (Engel and Chade, 2019; Engel et al., 2019). Like the injured heart, timely polarization towards an M2 phenotype promotes resolution of inflammation and optimal healing/regeneration in AKI (Cao et al., 2015).

### 3.2 Chronic injury

Macrophages also play a role in chronic kidney disease (CKD), which is most commonly caused by hypertension and/or metabolic disorders such as obesity and type 2 diabetes (T2D) (Hall et al., 2021). Hypertension causes renal injury via increased glomerular pressures as well as local and systemic increases in SNS/RAAS activation, which promote M1 macrophage polarization (Justin Rucker and Crowley, 2017). Another major feature of some forms of hypertension is increased sodium reabsorption, leading to increased interstitial sodium levels (Fehrenbach and Mattson, 2020; Ruggeri Barbaro et al., 2021). High salt (i.e., increased interstitial sodium levels) can act as a monocyte chemoattractant, and promotes M1 polarization via a mechanism, that is, not yet precisely understood (Willebrand and Kleinewietfeld, 2018; Ruggeri Barbaro et al., 2021). Both M1 and M2 macrophages, as well as intermediate phenotypes, contribute to renal dysfunction in CKD (Wen and Crowley, 2020; Cantero-Navarro et al., 2021). During CKD, DAMPs are continuously generated, and thus monocytes are continuously recruited from bone marrow reservoirs. These monocytes ultimately give rise to M1 macrophages, which promote parenchymal cell apoptosis and endothelial dysfunction, followed by M2 macrophages which promote tissue fibrosis.

## 4 Roles of conventional fuels during cardiorenal injury

**FIGURE 1:**
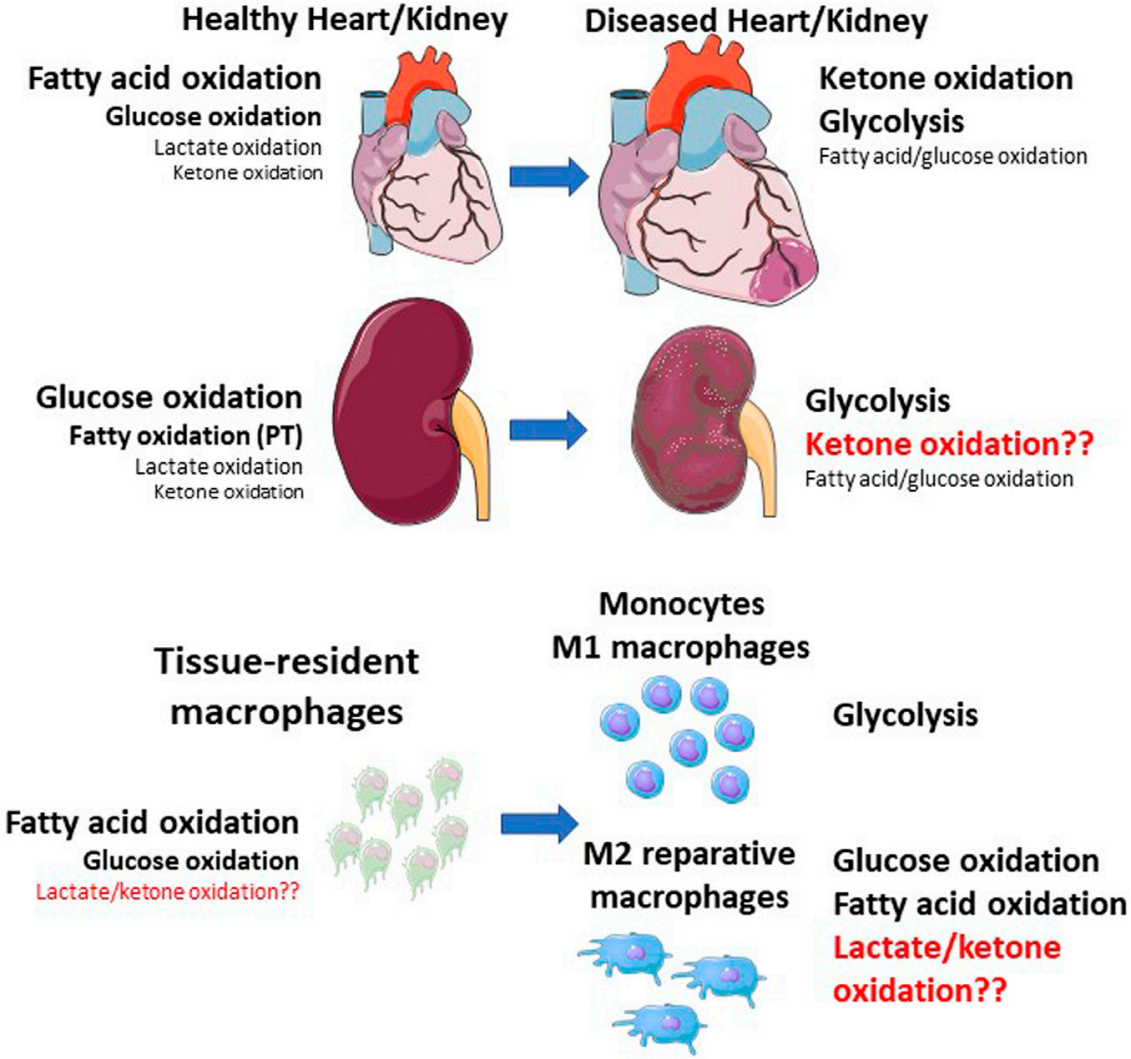
**Shifts in whole tissue and macrophage metabolic shifts during cardiorenal injury.** The healthy heart primarily relies on fatty acid oxidation, followed by glucose oxidation, with minor contributions from lactate and ketone oxidation. The kidney primarily relies on glucose oxidation, followed by fatty acid oxidation (mainly in the proximal tubule), with minor contributions from lactate and ketone oxidation. During cardiac injury, ketone oxidation and glycolysis are increased, while mitochondrial dysfunction results in impaired fatty acid and glucose oxidation. Macrophages within these tissues also shift their metabolic status. Resident macrophages rely on fatty acid and glucose oxidation, while monocytes and M1 macrophages primarily rely on glycolysis. M2 macrophages also primarily rely on glucose and fatty acid oxidation. Red text indicates unknown pathways. PT—proximal tubule.

Metabolic reprogramming is a hallmark of polarization between pro- and anti-inflammatory macrophages (Zhang et al., 2019a; Viola et al., 2019; Mouton et al., 2020; Mouton and Hall, 2020; Mouton et al., 2021). In this section we discuss ways in which different macrophage subsets utilize conventional fuels, such as glucose, fatty acids, and glutamine during cardiorenal injury, in the context of changes in cardiac and renal metabolism during health and disease (Figure 1).

### Glucose

Glucose metabolism is critical for normal cardiac and renal function. Next to fatty acids, glucose oxidation is a major source of ATP for the heart. The inner medullary nephrons of the kidneys also heavily rely on glucose (Figure 1). (Mizuno et al., 2017; Lopaschuk et al., 2020; Wen et al., 2021; Luong et al., 2022) The outer medulla of the nephron is a major source of gluconeogenesis, after the liver (Wen et al., 2021). In addition to the parenchymal cells of the heart and kidney, glucose is a critical fuel for both M1 and M2 macrophages. Glucose is initially metabolized through the glycolysis pathway, yielding two molecules of pyruvate. This pyruvate can then either be reduced to lactate, typically during hypoxic conditions, or taken up into mitochondria and oxidized in the tricarboxylic acid (TCA) cycle. In M1 macrophages, increased expression of glycolytic enzymes is mediated by the hypoxia-inducible factor-1 alpha (HIF-1α) transcription factor. HIF-1α is activated mainly by hypoxia, but also by inflammatory stimuli via the nuclear factor kappa-light-chain-enhancer of activated B cells (NF-κB) pathway (Viola et al., 2019).

Increased glucose uptake and glycolysis result in activation of the pentose phosphate pathway (PPP) via conversion of glucose-6-phosphate to ribulose-5-phosphate. The PPP produces ribose-5-phosphate for proliferating cells, as well as NADPH for pro-inflammatory processes such as the pathogen-killing oxidative burst and synthesis of pro-inflammatory lipid mediators (Viola et al., 2019). M2 macrophages also utilize glucose, mainly through glucose oxidation (aerobic glycolysis) (Tan et al., 2015; Viola et al., 2019). Inhibiting glucose oxidation prevents M2 polarization, while enhancing glucose oxidation through the pyruvate dehydrogenase complex attenuates lipopolysaccharide-induced inflammatory responses (Tan et al., 2015). However, glucose uptake is markedly increased in M1 compared to M2 macrophages, likely due to their reliance on glycolysis which produces much less ATP than full oxidation (Cho et al., 2022).

Altered glucose metabolism is a hallmark of polarized macrophages after cardiac injury (Mouton et al., 2018). Drastic upregulation of hypoxia responses and glycolytic genes are observed by RNA-sequencing in macrophages at day 1 after MI in mice (Mouton et al., 2018). This is followed by significant upregulation of mitochondrial pathways and oxidative phosphorylation at day 3, likely as a result of increased oxygen delivery from angiogenesis, which is associated with macrophage polarization toward a reparative M2 phenotype (Mouton et al., 2018). Transgenic studies have revealed that different HIF isoforms differentially regulate macrophage glucose and fatty acid metabolism after MI (DeBerge et al., 2021a). For example, HIF-1α upregulates glycolysis, while HIF-2α suppresses mitochondrial fatty acid oxidation to promote an inflammatory phenotype (DeBerge et al., 2021a). Further studies by the same group showed that the receptor tyrosine kinase AXL, which is involved in the clearance of apoptotic cells, is upregulated in macrophages during myocardial ischemia and promotes STAT1-mediated activation of HIF-1α, leading to increased glycolysis and inflammation (DeBerge et al., 2021b). This HIF-1α-mediated induction of glycolysis promotes cleavage and inactivation of MerTK, a major receptor involved in phagocytosing apoptotic cells, thus worsening cardiac repair (DeBerge et al., 2021b). Another study showed that administration of aminooxyacetic acid, an inhibitor of aspartate aminotransferase, inhibits glycolysis and M1 polarization by restoring the TCA cycle and decreasing levels of succinate (Zhao et al., 2020).

Glucose metabolism may also play a role in immunometabolic reprogramming during renal injury. Gene expression profiles from renal biopsies in patients with various inflammatory kidney disorders [i.e., systemic lupus nephritis (SLE)] demonstrate downregulation of TCA cycle and fatty acid oxidation genes, and upregulation of glycolytic and PPP genes which co-localized with inflammatory markers (Grayson et al., 2018). Similar results have been reported in a mouse model of autoimmune-associated nephritis, which induces glycolytic and inflammatory renal macrophage phenotypes, as well as downregulation of fatty acid oxidation genes (Jing et al., 2020). Furthermore, *in vivo* inhibition of glycolysis using 2-deoxyglucose attenuates renal inflammation and injury (Jing et al., 2020). In diabetic kidney disease, high glucose levels promote macrophage activation via HIF-1α activation mediated by TGF-β-activated kinase 1-binding protein-1 (TAB1), and inhibiting TAB1 alleviates kidney inflammation and disease progression (Zeng et al., 2020). In a model of unilateral ureteral obstruction, sphingosine-1-phosphate promotes macrophage glycolysis and inflammation via the sphingosine kinase 2 (Sphk2) pathway, suggesting that sphingolipid signaling may be a critical immunometabolic regulator in kidney injury (Ghosh et al., 2018).

Regulation of glucose metabolism in the kidney may depend on the local environment as the cortex has a high aerobic metabolic rate due to tubular sodium reabsorption, and thus a higher blood flow, while the medulla is more hypoxic (Basso et al., 2021). Tubular epithelial cells (TECs) are one of the major parenchymal cells of the kidney, and reprogram their metabolism from fatty acid oxidation to glycolysis in response to stress and inflammatory stimuli, similar to macrophages (van der Rijt et al., 2022). TECs can participate in immunometabolic cross-talk with macrophages, as extracellular vesicles secreted from TECs in diabetic mice promote macrophage glycolysis (Jia et al., 2022), suggesting that TECs and macrophages share similar metabolic reprogramming pathways and thus may be similarly affected by immunometabolic interventions (Li et al., 2021).

### 4.2 Fatty acids

Fatty acid metabolism is important for normal function of the heart and kidneys. At rest, the healthy heart and kidney cortex rely mainly on fatty acid oxidation (Figure 1). (Baverel et al., 1995; Lopaschuk et al., 2020; Dong et al., 2021; Wen et al., 2021) Macrophage metabolic reprogramming involves distinct changes in the mitochondrial TCA cycle, which is influenced by glucose and fatty acid oxidation (Zhang et al., 2019b; Viola et al., 2019). In inflammatory macrophages, the TCA cycle is truncated at key points to allow accumulation of key metabolites, in particular succinate and itaconate (Viola et al., 2019). In M2 macrophages, however, the TCA cycle is fully activated to generate NADH and FADH_2_ to fuel oxidative phosphorylation (OXPHOS). The fuel for macrophage mitochondrial OXPHOS is largely derived from beta-oxidation of fatty acids, which are taken up by circulating lipoproteins or as free fatty acids (Batista-Gonzalez et al., 2019). While fatty acid oxidation clearly supports M2 polarization, there is controversy as to whether the M2 macrophage phenotype relies on fatty acid oxidation, as some studies indicate that glucose or glutamine oxidation is sufficient to support the M2 phenotype (Batista-Gonzalez et al., 2019). However, in the context of cardiac injury, fatty acid oxidation appears to be important, as macrophages in the infarcted heart use fatty acids derived from engulfed dying cells to fuel mitochondrial OXPHOS and reparative gene expression (Zhang et al., 2019b).

### 4.3 Glutamine

Glutamine is the most abundant amino acid in the body and a critical fuel for all tissues, including macrophages, and decreased plasma glutamine levels are associated with cardiometabolic disease (Durante, 2019; Ren et al., 2019; Viola et al., 2019). Glutamine plays roles in inflammatory and reparative macrophages. M1 macrophages use glutamine as a precursor of glutamate, which is used to form gamma-aminobutyric acid (GABA) and fuels M1 polarization via replenishment of succinate and aspartate (Jiang et al., 2022). In M2 macrophages, glutamine can be used as an energy source via its conversion to alpha-ketoglutarate in the TCA cycle, which potentiates OXPHOS and is an activator of the histone demethylase Jmjd3 which epigenetically activates M2-associated genes (Liu et al., 2017). While both M1 and M2 macrophages use glutamine in different roles, M2 macrophages consume more glutamine and have higher levels of intracellular glutamine, and glutamine deprivation promotes M1 polarization (Palmieri et al., 2017; Ma et al., 2022).

Surprisingly few studies have investigated the role of macrophage glutamine metabolism in the context of cardiorenal injury. Several studies indicate that glutamine administration protects against cardiac injury in response to cardiotoxic cancer drugs (Shao et al., 2020), sepsis (Yin et al., 2014), severe burn (Yan et al., 2012), and ischemia/reperfusion (Liu et al., 2007), and renal inflammation and injury in response to sepsis (Su et al., 2021). However, glutamine metabolism appears to be a maladaptive phenomenon during pressure-overload induced heart failure, and blocking glutamine metabolism improves cardiac function in this model (Yoshikawa et al., 2022).

## 5 Potential roles of alternative fuels during cardiorenal injury

Although glucose, fatty acids, and glutamine are the conventional body fuels under steady state conditions, several tissues such as the heart and kidney can utilize alternative fuels during non-steady state and even pathological conditions. In this section, we discuss the contributions of alternative fuels, including ketones and lactate, to the macrophage phenotype during cardiorenal injury (Bellomo, 2002; Dong et al., 2021).

### 5.1 Ketones

Given the rise in popularity of low-carbohydrate/ketogenic diets, many recent studies have focused on the roles and potential therapeutic effects of the major ketone bodies, including beta-hydroxybutyrate (BHB) and acetoacetate (AA) (Abbasi, 2018). Both the heart and kidneys are capable of metabolizing ketones during starvation or consumption of ketogenic diets, and consume exogenously infused ketones in humans (Baverel et al., 1995; Mizuno et al., 2017; Puchalska and Crawford, 2017; Cuenoud et al., 2020; Lopaschuk et al., 2020). During injury, the heart and kidneys can increase ketone oxidation as an adaptation to chronic pathological stress (Figure 1). (Horton et al., 2019; Lopaschuk et al., 2020; Tomita et al., 2020) The kidneys play an important role in reabsorption of ketones, particularly in the proximal tubules which reabsorb close to 100% of filtered ketones during states of ketosis and thus conserve energy (Puchalska and Crawford, 2017; Palmer and Clegg, 2021).

Increased availability of ketones, especially via exogenous administration, protects against cardiac and renal dysfunction and injury in several preclinical models as well as in human patients. Chronic BHB infusion prevents pathological remodeling in murine and canine models of HF (Horton et al., 2019; Yurista et al., 2021), while acute BHB administration increases myocardial blood flow in healthy human subjects (Gormsen et al., 2017) and improves cardiac function in patients with HFrEF (Nielsen et al., 2019). Oral ketone administration also improves cardiac function and inflammation in a porcine model of MI (Yurista et al., 2021). Exogenous ketones can protect against different types of renal injury, including ischemia-reperfusion (Tajima et al., 2019), hypertension (Ishimwe et al., 2020), salt sensitivity (Chakraborty et al., 2018), and diabetes (Tomita et al., 2020). The roles of ketogenic diets on renal function and disease progression are less clear. Ketogenic diets have been postulated to protect against kidney disease by attenuating inflammation, oxidative stress, and mitochondrial dysfunction; however, these assumptions are largely based on studies in which exogenous ketones are administered (Kundu et al., 2022). In rodent models, ketogenic diets have been reported to ameliorate acute ischemic kidney injury (Rojas-Morales et al., 2022). However, another study showed that a short-term (4 weeks) ketogenic diet exacerbated renal injury in spontaneously-hypertensive (SH) rats, which was associated with impaired glucose tolerance and increased activation of the renin-angiotensin-aldosterone system (Jia et al., 2021). However, no injury was noted in the wild-type group, indicating that SH rats may not tolerate a ketogenic diet well. Overall, more studies are needed to evaluate the impact of ketogenic diet on cardiovascular disease.

**FIGURE 2:**
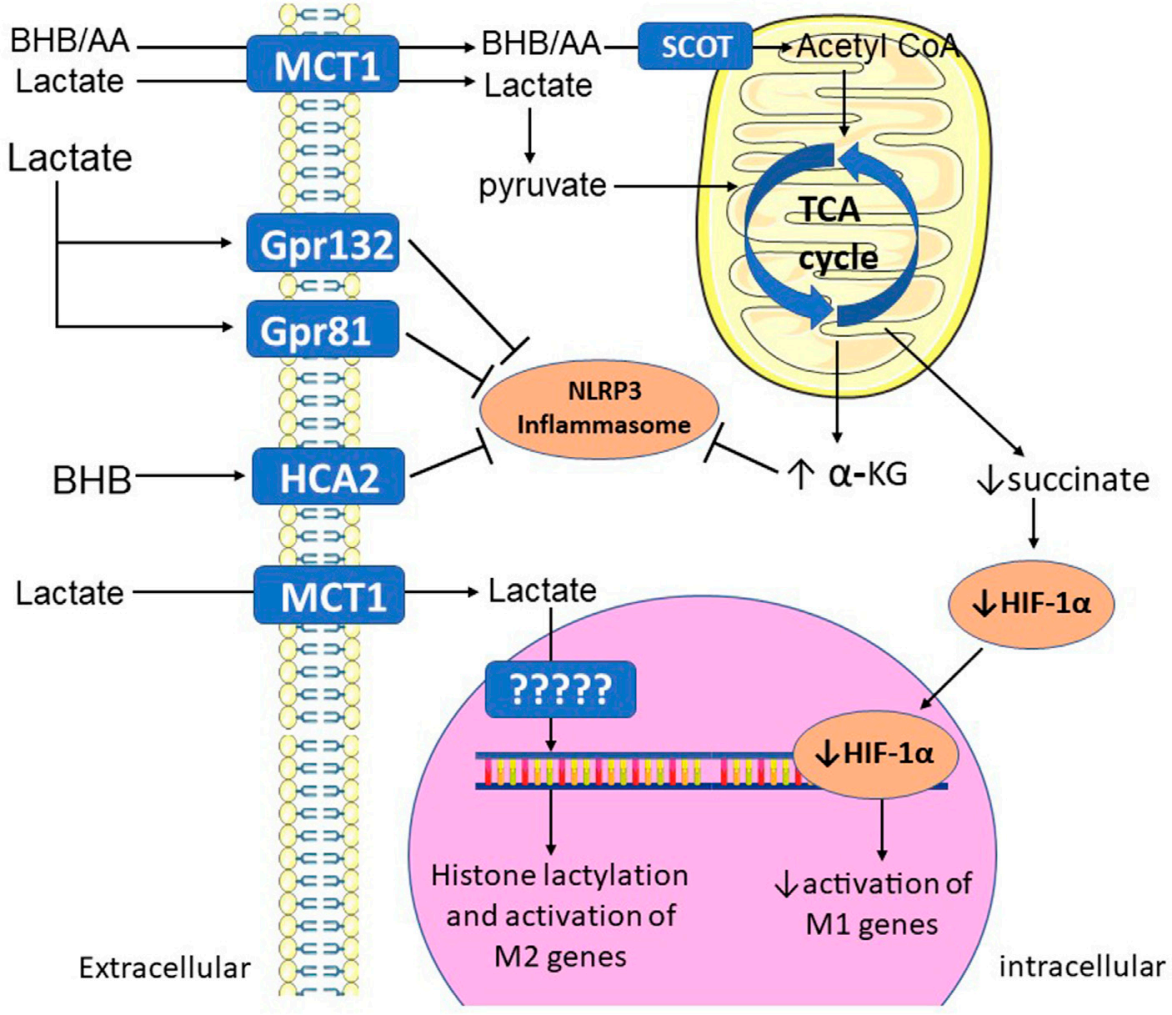
**Mechanisms by which alternative fuels promote M2 polarization.** BHB and AA (ketones), and lactate, are taken up by MCT1 in the plasma membrane, then oxidized in the mitochondrial TCA cycle. Increased TCA cycle activity increases levels of alpha-KG, which blocks NLRP3 inflammasome activation. Full TCA cycle activation also prevents accumulation of succinate, decreasing its activation of HIF-1α and its activation of M1 genes. Extracellular lactate and BHB can also bind to membrane G-protein coupled receptors, which activate intracellular signaling pathways that block inflammasome activation. Lactate can also be used to covalently modify histone proteins (lactylation), leading to activation of M2 genes. α-KG—alpha-ketoglutarate; AA—acetoacetate; BHB—beta-hydroxybutyrate; Gpr—G-protein receptor; HCA2—hydroxycarboxylic acid receptor 2; HIF-1α—hypoxia-inducible factor 1-alpha; MCT1—monocarboxylate transporter 1; SCOT—succinyl-CoA:3-ketoacid-coenzyme A transferase; TCA—tricarboxylic acid.

Ketones have potent anti-inflammatory roles in macrophages (Figure 2). (Youm et al., 2015; Puchalska et al., 2019) BHB ketone therapy lessens macrophage-mediated inflammation and disease severity in patients with colitis (Huang et al., 2022). BHB directly blocks inflammasome formation and macrophage activation, which is independent of its energetic capacity (Youm et al., 2015). In a mouse model of stroke, BHB limits neuronal injury by activating a G-protein coupled receptor (HCA2) to promote anti-inflammatory macrophage polarization (Rahman et al., 2014). On the other hand, liver fibrosis induced by a fibrogenic diet is exacerbated in mice lacking the enzyme that oxidizes ketone bodies to acetyl-CoA (SCOT) in macrophages (Puchalska et al., 2019), indicating that activation of signaling pathways and metabolism are important for the anti-inflammatory effects of BHB depending on the type of injury and tissue. In summary, ketones exert anti-inflammatory actions in macrophages via both metabolic and non-metabolic pathways.

### 5.2 Lactate

Lactate is a byproduct of anaerobic glycolysis, via reduction of pyruvate by lactate dehydrogenase. Far from being a waste product, lactate has a multitude of metabolic and signaling roles throughout the body. Both the heart and kidney, specifically the renal cortex, consume and metabolize a large amount of lactate at rest (Figure 1), and lactate becomes one of the major fuels for the heart during intense exercise (Bellomo, 2002; Brooks, 2021; Dong et al., 2021; Wen et al., 2021). The renal medulla, on the other hand, produces large amounts of lactate via glycolysis, which is removed by the cortex (Bellomo, 2002). The kidneys, as well as the liver, also play a role in removing excess lactate from the circulation, with most being metabolized and only a small amount being excreted (Bellomo, 2002). Under normal blood lactate levels, most of the filtered lactate is reabsorbed in the proximal tubules (Bellomo, 2002). Lactate levels increase in the pathologically hypertrophied heart and the infarcted heart as a result of metabolic impairments (Lewis et al., 2018; Dai et al., 2020). In the infarcted heart, this lactate accumulation is a result of infiltrating glycolytic monocytes (Lewis et al., 2018), and lactate accumulates in the acutely injured kidney as a result of increased glycolysis in TECs (Shen et al., 2020).

Similar to ketones, lactate also has potent anti-inflammatory roles in macrophages (Figure 2). (Caslin et al., 2021; Zhou et al., 2022a) By accumulating as a result of increased glycolysis in inflamed tissues, lactate appears to exert negative feedback on the inflammatory response, promoting tissue macrophages towards an M2/pro-reparative phenotype and accelerating wound healing (Zhang et al., 2020; Jha et al., 2021; Zhou et al., 2022b; Wang et al., 2022). A prime example of this is in skeletal muscle ischemia, in which endothelial-derived lactate promotes M2 polarization and regeneration of the injured tissue, which is lost when the lactate transporter MCT1 is genetically deleted in macrophages (Zhang et al., 2020). Consistent with this finding, exogenous lactate appears to accelerate inflammation resolution and wound healing. After MI, lactate treatment attenuates inflammation and fibrosis, promotes M2 polarization, and improves cardiac function (Zhang et al., 2021). Acute lactate administration improves cardiac function in acute heart failure patients (Nalos et al., 2014), and improves short and long-term outcomes in patients with traumatic brain injury (Ichai et al., 2009). In adult pigs, lactate improves renal vascular thrombosis and renal function (glomerular filtration rate) in a model of endotoxemia (Duburcq et al., 2018). However, lactate appears to exacerbate complications associated with sepsis (Yang et al., 2022a; Yang et al., 2022b). Furthermore, lactate’s wound healing abilities may present a double-edged sword, as lactate has been shown to promote a myofibroblast phenotype in injured kidneys (Shen et al., 2020). While myofibroblasts play a role in acute wound healing in renal injury, they can also promote tissue fibrosis when chronically activated (Zhao et al., 2022). Thus, lactate therapies aimed at reducing macrophage-mediated inflammation should consider the impact on fibrotic pathways.

Like ketones, the anti-inflammatory mechanisms of lactate appear to be mediated by metabolic and non-metabolic mechanisms (Manoharan et al., 2021). Macrophages are capable of directly metabolizing lactate, and macrophages polarized to an M2 phenotype by IL-4 undergo a metabolic switch to increase lactate oxidation, leading to increased TCA cycle activity and OXPHOS (Geeraerts et al., 2021; Noe et al., 2021). Lactate can also promote M2 polarization by binding to G-protein coupled receptors in the plasma membrane, including Gpr132 and Gpr81 (Chen et al., 2017; Yang et al., 2020). Recent studies have also found a role for lactate in promoting epigenetic modifications of histone proteins, termed lactylation (Liu et al., 2022). In macrophages, lactylation promotes activation of genes involved in wound healing/repair, such as Arg*1* (Zhang et al., 2019c). In a mouse model of MI, the macrophage M2 genes *Il10* and *Vegfa* were found to have lactylation regulatory sites and blocking lactylation of these genes exacerbated post-MI inflammation and impaired angiogenesis (Wang et al., 2022).

In summary, cardiac and renal injury are characterized by lactate accumulation in the injured tissue, which may drive activation of reparative macrophage phenotypes to promote tissue healing. However, lactate may exacerbate injury during certain disease states, such as sepsis. Thus, more studies are needed to assess the role of lactate and macrophage phenotypes across different disease states.

## 6 Perturbations of immunometabolic pathways during metabolic syndrome

Obesity and diabetes represent two of the major factors that contribute to metabolic dysfunction in patients with cardiorenal disease (Ray et al., 2016). Obesity and diabetes promote macrophage-mediated inflammation by altering metabolic substrates such as glucose and fatty acids, thus directly affecting macrophage metabolism via changes in available fuels (Table 1) (Appari et al., 2018; Mouton et al., 2020). In this section, we discuss the potential effects of obesity and diabetes on macrophage metabolism, and the ramifications for cardiorenal diseases.

**TABLE 1:** Mechanisms of M1 macrophage activation in obese/T2D individuals.

Non-metabolic	Mechanism	Metabolic	Mechanism
Tissue hypoxia	HIF-1α activation	Hyperglycemia	Mitochondrial dysfunction; AGEs activation of AGE receptor (RANGE), NF-κB
Tissue/microvascular injury	Chemokine/cytokine-mediated activation of monocytes	Hypertriglyceridemia	Activation of TLR4 by long chain saturated fatty acids
↑leptin	Activation of Intracellular signalling pathways via leptin receptor (LepR)	↑Omega-6/Omega-3 fatty acid ratio	↑production of pro-inflammatory eicosanoids
↓adiponectin	Loss of anti-inflammatory actions via the AdipoR receptor	↓Ketogenesis	***Unknown***
		↑plasma lactate levels	

### 6.1 Obesity

Over 10% of adults worldwide and 40% of adults in the United States are considered obese (BMI >30 kg/m^2^), which is a risk factor for type 2 diabetes (T2D), hypertension, cardiovascular disease, and chronic kidney disease (Khwatenge et al., 2021). Obesity is a major risk factor for hypertension, as ∼70% of individuals with obesity are hypertensive, which causes cardiorenal dysfunction and injury (Hall et al., 2019; Mouton et al., 2020; Hall et al., 2021). Furthermore, obesity can cause volume overload of the heart due to increases in blood volume and venous return, leading to eccentric hypertrophy (Hall et al., 2021). The incidence of HFpEF is increased in obesity, which is linked to an inflammatory phenotype (Sabbah et al., 2020; Hall et al., 2021). In the kidney, obesity causes injury and hypertension by physical compression due to increased visceral and renal sinus adipose tissue, as well as activation of the sympathetic nervous system and renin-angiotensin-aldosterone system, leading to increased sodium reabsorption (Hall et al., 2019; Hall et al., 2021).

Obesity is a major driver of chronic inflammation linked to macrophage activation (Appari et al., 2018). Even in “metabolically healthy” obese individuals, there are increased levels of circulating pro-inflammatory monocytes compared to lean control subjects (Christou et al., 2019). The most direct way in which obesity promotes inflammation is via proliferation and activation of adipose tissue macrophages (ATMs), which secrete pro-inflammatory cytokines that activate inflammatory pathways in other organs and tissues, leading to systemic inflammation (Appari et al., 2018). Obesity is characterized by increases in circulating free fatty acids and changes in the fatty acid profile that favor long-chain saturated fatty acids such as palmitate. These fatty acids activate inflammatory macrophage pathways via toll-like receptor 4 (TLR4) signaling. Also, high levels of oxidized phospholipids and ceramides activate inflammatory intracellular signaling cascades (Dahik et al., 2020; Sharma et al., 2020; Xu et al., 2022).

Despite increased availability of fatty acids which fuel OXPHOS, ATMs from obese mice prefer glycolysis due to adipose tissue hypoxia, which activates HIF-1α-mediated glycolytic metabolism (Serbulea et al., 2018; Sharma et al., 2020). ATMs shift to storing lipids rather than oxidizing them during obesity (Mooli and Ramakrishnan, 2022). Increased levels of omega-6/omega-3 fatty acids and arachidonic acid are characteristic of obesity (Simopoulos, 2016; Rombaldova et al., 2017; Xu et al., 2021), and can shift the synthesis of lipid mediators towards pro-inflammatory, rather than pro-resolving (Rombaldova et al., 2017; Xu et al., 2021).

Obesity may also promote macrophage activation via changes in adipokines (Mouton et al., 2020). Leptin is a major satiety hormone secreted by adipose tissue and is often elevated in obese patients (Mouton et al., 2020). Leptin also promotes macrophage M1 polarization (Mouton et al., 2020). On the other hand, obesity decreases levels of adiponectin, an anti-inflammatory adipokine (Mouton et al., 2020).

Obesity impairs ketogenesis in the liver, mainly through increased insulin levels that counteract ketogenesis as well as continuous supplies of glucose and fatty acids either obtained from the diet or saturated glycogen and adipose stores (Vice et al., 2005; Puchalska and Crawford, 2017; Mooli and Ramakrishnan, 2022). Obesity also impairs ketone oxidation in skeletal muscles of female patients with obesity (Vice et al., 2005). On the other hand, obesity was recently reported to increase skeletal muscle ketone oxidation in mice (Al Batran et al., 2020). Thus, the impact of obesity on cardiac and renal ketone metabolism remains unclear (Selvaraj et al., 2020; Kolwicz, 2021).

Obesity may affect lactate metabolism, although the data are less clear. Plasma lactate levels are higher in individuals with obesity, possibly as a result of increased anaerobic glycolysis in white adipocytes (Lovejoy et al., 1992; Chondronikola et al., 2018; Krycer et al., 2020; Lagarde et al., 2021). Recent studies have shed light on the role of adipose tissue-derived lactate in macrophage metabolic remodeling. Increased levels of adipocyte-derived lactate promoted M1 polarization in ATMs during obesity, suggesting a unique role for lactate in ATMs (Feng et al., 2022; Lin et al., 2022). On the other hand, chronic administration of lactate attenuated adipose tissue inflammation, M1 polarization, and insulin resistance in mice on a high-fat diet, indicating that exogenously administered lactate may exert its effects via different mechanisms than endogenously-produced lactate by ATMs (Cai et al., 2022). Given that lactate administration appears to be protective after MI and renal injury (Duburcq et al., 2018; Zhang et al., 2021), as previously discussed, further studies are needed to determine the role of lactate during obesity-related cardiorenal injury.

### 6.2 Diabetes

Type II diabetes (T2D) is an increasingly prevalent metabolic disorder, and is closely associated with obesity (Mouton et al., 2020). T2D is mainly characterized by systemic insulin resistance, leading to hyperinsulinemia and hyperglycemia (Mouton et al., 2020; da Silva et al., 2020). Diabetic cardiomyopathy (DCM) is characterized by ventricular wall thinning and impaired contractile function as well as cardiac inflammation (Kaur et al., 2021). T2D can also indirectly impair cardiac function by drastically increasing the risk of developing hypertension, coronary artery disease, and MI. Patients with T2D are at greater risk of MI and T2D exacerbates post-MI inflammation while impairing wound healing and worsening cardiac function, and increasing mortality (von Bibra and John Sutton, 2011; Yap et al., 2019; Yang et al., 2019; Rawal et al., 2019; Paolisso et al., 2021; Marfella et al., 2003; Terlecki et al., 2013). T2D is also a major risk factor for renal injury and dysfunction, and diabetic kidney disease (DKD) is characterized by glomerular and tubulointerstitial injury and inflammation (Maric and Hall, 2011). The proximal tubule of the nephron, as the major site of glucose reabsorption, is particularly sensitive to elevated glucose levels, and undergoes significant hypertrophy during the early stages of DKD due to increased tubular reabsorption (Barrera-Chimal and Jaisser, 2020). T2D also dramatically increases the development of renal injury and failure during hypertension (Wang et al., 2020).

T2D interacts with macrophage immunometabolic pathways in several ways. In addition to the mechanisms previously discussed for obesity, the most obvious way in which T2D promotes M1 polarization is via hyperglycemia, which exposes macrophages to supranormal levels of glucose (Mouton et al., 2020; Orliaguet et al., 2020). Exposure to high glucose either *in vivo* or *in vitro* appears to decrease macrophage glycolysis, indicating a non-glycolytic role for high glucose levels in promoting M1 polarization (Pavlou et al., 2018; Ayala et al., 2019; Matsuura et al., 2022). High glucose levels may instead promote M1 polarization via accumulation of advanced glycation end products (AGEs), which are recognized by receptors for AGEs (RAGEs), which activate the NF-κB pathway (Jin et al., 2015; Bezold et al., 2019). Patients with T2D typically suffer from hyperlipidemia due to impaired insulin signaling in adipose tissues, leading to macrophage lipotoxicity (Li et al., 2022a).

T2D is associated with alterations in ketone metabolism. Hyperglycemia has been shown to impair hepatic ketogenesis (Fletcher et al., 2019; Chung et al., 2022). However, ketones are often elevated in uncontrolled T2D, perhaps due to impaired ability to metabolize them. Glucose and fatty acids, which are elevated in T2D (Luong et al., 2022), compete with ketones in the mitochondria, thus lowering their utilization (Abdul Kadir et al., 2020; Garcia et al., 2020; Petrick et al., 2020). Contrary to the failing heart in non-T2D subjects, which increases ketone oxidation, hearts from subjects with T2D show impairments in ketone metabolism due to downregulation of ketolytic enzymes (Turko et al., 2001; Brahma et al., 2020). Given that ketones are more efficiently oxidized in the mitochondria and boost mitochondrial function during T2D (Parry et al., 2018; Chung et al., 2022), ketone therapy may restore mitochondrial function during T2D.

One of the most popular therapeutics currently used to manage glucose levels in T2D is sodium-glucose transporter-2 (SGLT2) inhibitors, which increase glucose excretion by the kidney (Saucedo-Orozco et al., 2022). In addition to lessening renal injury in T2D, these drugs are efficacious in treating heart failure in non-diabetic patients (Saucedo-Orozco et al., 2022). One of the mechanisms by which SGLT2 inhibitors are thought to exert their cardiorenal-protective effects is by elevating levels of ketone bodies, which reduce macrophage M1 polarization by activating the NLRP3 inflammasome (Kim et al., 2020; Saucedo-Orozco et al., 2022). Empagliflozin, one of the major SGLT2 inhibitors, increases ketolytic enzyme expression and ketone oxidation in the heart after MI (Yurista et al., 2019). Contrary to the heart, the kidney energetic profile has been reported to shift from fatty acid oxidation to ketone oxidation in a rat model of diabetic nephropathy (Tomita et al., 2020). However, administration of ketones or raising ketones with empagliflozin ameliorated renal injury and dysfunction in a ketogenic-dependent manner, indicating that the switch to ketolytic metabolism is not sufficient to maintain renal function during T2D (Tomita et al., 2020). Whether T2D directly impairs macrophage ketone metabolism remains to be studied.

T2D also affects lactate metabolism. T2D decreases lactate uptake in human hearts (Mizuno et al., 2017) and decreases lactate oxidation in rat hearts, concomitant with decreased glucose oxidation and increased fatty acid oxidation (Chatham et al., 1999; Dong et al., 2021). High glucose levels have also been shown to affect lactate metabolism in non-macrophage cell types *in vitro*, such as impairing lactate oxidation in myotubes (Lund et al., 2019) and decreasing Mct1 expression in tumor cells (De Saedeleer et al., 2014). However, the consequence of impaired lactate oxidation in the T2D heart is still unclear. The role of lactate metabolism in DKD is even less clear. The diabetic kidney has been reported to shift from glucose oxidation to glycolytic metabolism even in the presence of oxygen (Warburg effect), leading to accumulation of lactate (Zhang et al., 2018). Given the fact that mitochondrial dysfunction and impaired OXPHOS is a hallmark of DKD, it could be speculated that along with the shift towards glycolysis, lactate oxidation may be impaired, leading to systemic and local increases in lactate (Zhang et al., 2018). As discussed previously, lactate promotes a metabolic shift in fibroblasts to promote a myofibroblast phenotype, leading to renal fibrosis (Ding et al., 2017; Shen et al., 2020) and linking increased lactate in DKD to aggravated injury (Laustsen et al., 2019; Lee et al., 2022). However, the role of macrophage lactate metabolism in T2D remains to be determined.

## 7 The central nervous system (CNS) and macrophage activation

The CNS plays critical roles in regulating full body metabolism, especially via inputs to the autonomic nervous system from the hypothalamus (Myers and Olson, 2012). Recent evidence suggests that the CNS may also play an important role in modulating macrophage activation. The autonomic nervous system regulates immune responses via sympathetic and parasympathetic inputs to the spleen and bone marrow, (Baez-Pagan et al., 2015; Rocha-Resende et al., 2021), as well as inflammatory responses during cardiac and renal injury (Inoue, 2021; Rocha-Resende et al., 2021). In general, sympathetic activation leading to release of norepinephrine modulates macrophage phenotypes via activation of alpha-adrenergic receptors, which promotes M1 phenotypes, while activation of beta-adrenergic receptors promotes M2 phenotyptes, although there are some exceptions (Freire et al., 2022). Meanwhile, parasympathetic activation of α7 nicotinic acetylcholine receptor (α 7nAChR) by acetylcholine mediates anti-inflammatory responses in macrophages, mainly via inhibition of signaling pathways including NF-κB, STAT3, and heme oxygenase (Baez-Pagan et al., 2015). Increasing parasympathetic activity using drugs that inhibit acetylcholinesterase (e.g., donepezil) protects the heart against cardiac remodeling and improves prognosis in heart failure induced by ischemia/reperfusion injury (Li et al., 2020; Li et al., 2022b). This cardiac protective effect can be prevented by blockade of peripheral α7nAChR (183), indicating that the α7nAChR is also critical for the cardiac protection conferred by the cholinergic anti-inflammatory pathway. However, the direct involvement of α7nAChR in macrophages in cardiac protection is still unclear. Stimulation of the vagus nerve also attenuates renal dysfunction and injury after acute kidney injury induced by bilateral ischemia/reperfusion (Inoue et al., 2016; Inoue, 2021). During CKD, chronic sympathetic activation overrides the cholinergic anti-inflammatory system, leading to chronic inflammation which may worsen renal injury (Hilderman and Bruchfeld, 2020). Pilot studies in hemodialysis patients implanted with cervical vagal nerve stimulation implants have shown promise for treating chronic inflammation during CKD (Hilderman and Bruchfeld, 2020).

Leptin may be a critical mediator linking neural pathways to macrophage activation and metabolism. As mentioned previously, leptin modulates macrophage activity (Mouton et al., 2020). Since the leptin-melanocortin system is an important regulator of autonomic activity and substrate metabolism in peripheral tissues, it is possible that activation of this pathway in the CNS affects macrophage phenotypes (Okamoto et al., 2000; Tanaka et al., 2010). A couple of studies have demonstrated that CNS actions of leptin mediate immune responses to infectious insults (Tschop et al., 2010; Flatow et al., 2017). In a model of renal injury induced by unilateral ureteral obstruction, central leptin administration exacerbated renal macrophage infiltration in obese diabetic mice (Tanaka et al., 2010). However, we recently showed that activation of the CNS leptin-melanocortin system protects the heart against progressive heart failure and improves cardiac function after ischemic injury (Gava et al., 2021; Omoto et al., 2022). Given these seemingly contradictory findings, additional studies are needed to examine whether the CNS modulates macrophage metabolism and inflammation in cardiorenal disease via leptin signaling or other pathways.

## 8 Concluding remarks

Macrophage polarization plays a dichotomous role in tissue repair/regeneration and loss of parenchymal cell function/fibrosis during cardiorenal diseases. The field of immunometabolism has emerged as a promising therapeutic avenue for cardiorenal diseases, particularly in patients with underlying metabolic impairments. While we have discussed roles for different metabolic substrates and metabolic shifts in the parenchymal cells of the heart and kidneys, little is still known about macrophage metabolism during diseases, particularly with regards to different macrophage subsets. Metabolism of alternative fuels, particularly ketones and lactate, has become increasingly appreciated as playing a critical role during tissue injury and remodeling, particularly via actions on macrophage-mediated inflammation. Targeting macrophage-mediated inflammation and tissue injury is a promising therapeutic avenue for treating cardiac and renal disease. Multiple studies have demonstrated that macrophage activation and phenotype switching are mediated by metabolic pathways during cardiorenal injury in preclinical models. However, the mechanisms by which metabolic disorders, such as obesity and T2D which affect the majority of patients with CVD, cause macrophage activation remains unclear. Overall understanding of endogenous regulation of these alternative metabolic pathways in macrophages and their resident tissues is required to fully optimize therapeutic options, particularly in at-risk individuals.
